# 

**Published:** 1868-10

**Authors:** 


CHANGE OF NUMBER! In consequence of there being two numbers on West Fayette Street, causing some confusion, we have rectified it by changing to "86," which is our right number.

☞ Same place, only a change of Number. ☜

